# Exact association test for small size sequencing data

**DOI:** 10.1186/s12920-018-0344-z

**Published:** 2018-04-20

**Authors:** Joowon Lee, Seungyeoun Lee, Jin-Young Jang, Taesung Park

**Affiliations:** 1 Department of Statistics, Seoul National University, Seoul, South Korea; 2 Department of Applied Statistics, Sejong University, Seoul, South Korea; 3 Department of Surgery, Seoul National University College of Medicine, Seoul, South Korea

**Keywords:** NGS data analysis, Small size sequencing data, Association study, CMH statistic, IPMN, Fisher’s exact test

## Abstract

**Background:**

Recent statistical methods for next generation sequencing (NGS) data have been successfully applied to identifying rare genetic variants associated with certain diseases. However, most commonly used methods (e.g., burden tests and variance-component tests) rely on large sample sizes. Notwithstanding, due to its-still high cost, NGS data is generally restricted to small sample sizes, that cannot be analyzed by most existing methods.

**Methods:**

In this work, we propose a new exact association test for sequencing data that does not require a large sample approximation, which is applicable to both common and rare variants. Our method, based on the Generalized Cochran-Mantel-Haenszel (GCMH) statistic, was applied to NGS datasets from intraductal papillary mucinous neoplasm (IPMN) patients. IPMN is a unique pancreatic cancer subtype that can turn into an invasive and hard-to-treat metastatic disease.

**Results:**

Application of our method to IPMN data successfully identified susceptible genes associated with progression of IPMN to pancreatic cancer.

**Conclusions:**

Our method is expected to identify disease-associated genetic variants more successfully, and corresponding signal pathways, improving our understanding of specific disease’s etiology and prognosis.

## Background

Many genetic studies, such as genome-wide association studies (GWAS), have successfully identified genetic variants associated with complex human traits and diseases [1]. However, GWAS focus mainly on common variants with minor allele frequencies (MAF) greater than 0.05. Thus, loci with MAF < 0.05 are omitted, even though such “rare variants” may substantially contribute to disease heritability [2, 3]. The recent application of next generation sequencing (NGS) technology has put large-scale investigation of rare variants within reach [4]. Thus, from large sample sizes, researchers can uncover novel rare genetic variants (i.e., those having MAFs between 0.01 and 0.05) that have important associations with complex diseases [3].

To date, various statistical methods and strategies have been developed to test disease associations of rare genetic variants. Burden tests, which were earlier tests for rare variants, aggregate information from all rare variants, in a specific genomic region, into a single summary variable [5, 6]. Different types of burden tests have been proposed, using various genetic scores assigned to the rare variants. For example, the cohort allelic sum test (CAST) collapses genotypes across all variants, such that an individual is coded as 1, if a rare allele is present at any of the variant sites; otherwise, it is coded as 0 [6]. However, this approach may not fully reflect the effect emerging from the complex ensemble of multiple rare variants, because it only uses the information from the presence of rare variants within a specific genomic region.

The combined multivariate and collapsing (CMC) method divides rare variants into multiple classes, based on their MAFs, by collapsing each group, using CAST, and then applying multivariate tests such as Hotelling’s T-test [5]. However, these burden tests are powerful only if most rare variants are causal, and have effects in the same direction (i.e., increase or decrease the phenotype). In other words, the existence of variants whose effects are in different directions can reduce power substantially. To overcome this limitation, several variance-component (VC) tests, based on regression models, have been proposed. The Sequence Kernel Association Test (SKAT), a widely used score-based VC test, has been shown to successfully detect multiple directional contributions from different classes of single nucleotide polymorphisms (SNPs) [7].

Both burden and VC tests for rare variants are based on asymptotic tests, assuming that the sample size is large enough. Due to the still-high cost of NGS, however, sequencing data is often available only from small sample sizes. These existing methods are not appropriate to handle NGS data from small sample sizes. Instead, the SKAT method needs to be modified by renormalizing moments of test statistics [8].

In this study, we propose a new approach that does not rely on the asymptotic distribution for the NGS data with small samples. We call this new method the *Exact Association Test (EXAT)*. EXAT is conceptually based upon the Fisher’s exact test, which is commonly used for testing for independence, using 2 × 2 contingency tables, with small samples. A key underlying assumption of Fisher’s exact test is that the four marginal sums are fixed. Under this assumption, the first cell frequency follows a hypergeometric distribution, under the null hypothesis of independence. To that end, the Cochran-Mantel-Haenszel (CMH) statistic was developed to extend Fisher’s exact test beyond stratified 2 × 2 contingency tables, for testing the conditional independence between two categorical variables, that are in turn, conditioned by a third categorical variable [9, 10]. The generalized Cochran-Mantel-Haenszel (GCMH) statistic is an extension of CMH for stratified J × K contingency tables [9].

For a specific gene, NGS data can be represented by a sequence of contingency tables. The strata variable corresponds to the subject, the row variable does to the single nucleotide variant (SNV), and the column depicts the genotypes which represent the number of minor alleles (0, 1, or 2). For example, suppose that a gene contains *t* SNVs. Then, the NGS data from *n* individuals can be summarized into *n* × *t* × 3 contingency table, upon which the GCMH statistic can be applied. Note that this GCMH statistic is used for testing independence between SNVs and the number of minor alleles. That is, it tests whether *t* SNVs have similar distributions, in terms of MAFs. However, this GCMH does not provide any information about the gene’s association with disease status, e.g., case and control. Thus, we propose deriving the GCMH statistic separately from the case and control groups, and using the difference or ratio as a test statistic. If these two GCMH statistics differ greatly between case and control groups, then the gene should be strongly associated with disease status.

In the Methods section, we provide a detailed description of the EXAT statistic, and summarize how to compute *p*-values for significance testing. We then apply our EXAT to the analysis of targeted sequencing data from intraductal papillary mucinous neoplasms (IPMNs, a type of pancreatic ductal tumor)(PMID: 27865286). IPMN is a unique pancreatic neoplasm that can become an invasive, metastatic, and hard-to-treat pancreatic cancer [11]. Through this application, we demonstrate that our proposed EXAT method can successfully identify susceptible genes associated with the progression of IPMN to pancreatic cancer.

## Methods

### Materials

All human subject studies were approved by the Institutional Review Board of Seoul National University Hospital. Surgical paraffin-embedded IPMN samples, from 44 subjects, were obtained from Seoul National University Hospital. These subjects consisted of 21 cases of high grade (just before developing pancreatic cancer) and 23 controls of low grade (benign tumor). From both tumor groups, DNA was extracted and subjected to targeted sequencing, using the Illumina NextSeq500 platform.

The demographic and clinical characteristics of the 44 subjects are shown in Table 1. Categorical variables were compared using the χ^2^ test or Fisher’s exact test between case and control groups. Continuous variables were compared using Student’s t test or Wilcoxon’s rank sum test. Except *Mural Nodule* and *Invasiveness,* there were no significant differences between case and control groups. *Mural Nodule* is known as a potential predictor of malignant neoplasm [12], and *Invasiveness* presents an invasive status.

**Table 1:** Demographic and clinical characteristics of study patients at baseline

	Total (*n* = 44)	Case (*n* = 21)	Control (*n* = 23)	*P* − value
Continuous variables	Mean (SD)			
Age	64.57 (8.3)	64 (9.4)	65.09 (7.2)	0.668
CEA	2.46 (2.6)	2.90 (3.4)	2.04 (1.4)	0.293
CA19–9	60.61 (280.9)	2.89 (400.2)	2.04 (11.0)	0.061
Categorical variables	Frequency			
Sex (M:F)	28:16	15:6	13:10	0.476
Invasiveness ratio (Invasive: Noninvasive)	10:34	9:12	1:22	0.003
Mural Nodule (Yes: No)	19:24	13:8	6:16	0.032
Recurrence (Yes: No)	34:9	4:17	0:22	0.044
Survival (Yes: No)	34:9	17:4	17:5	1

From each patient, we obtained targeted sequencing data for 411 genes, known to be related to cancer in general, but not necessarily pancreatic cancer. The total number of SNVs was 8325, and the number of SNVs in a gene ranged from 1 to 188, with a median of 15.

### Methods

#### Data structure

First, we constructed a stratified categorical data as follows. For a given gene with *t* SNVs, we defined a *t* × 3 contingency table for each subject, where the rows and columns represent the SNVs for a specific gene, and the number of minor alleles, respectively. More precisely, for subject *i*, and a specific gene with *t* SNVs, the corresponding *t* × 3 contingency table was constructed, as shown in Table 2. Note that the cell count, *n*_*ijk*_,has a value of 1, if the subject *i* has a minor allele count *k*, at SNV *j*, for *i* = 1, ⋯, *n*,  *j* = 1, ⋯, *t*,  *k* = 0, 1, 2.

**Table 2:** Stratum representing subject *i*, for a specific gene, with *t* SNVs

SNV	Number of minor alleles			Total
	0	1	2	
1	*n*_*i*10_	*n*_*i*11_	*n*_*i*12_	1
⋮	⋮	⋮	⋮	1
*t*	*n*_*it*0_	*n*_*it*1_	*n*_*it*2_	1
Total	*n*_*i*. 0_	*n*_*i*. 1_	*n*_*i*. 2_	*t*

**Fig. 1:**
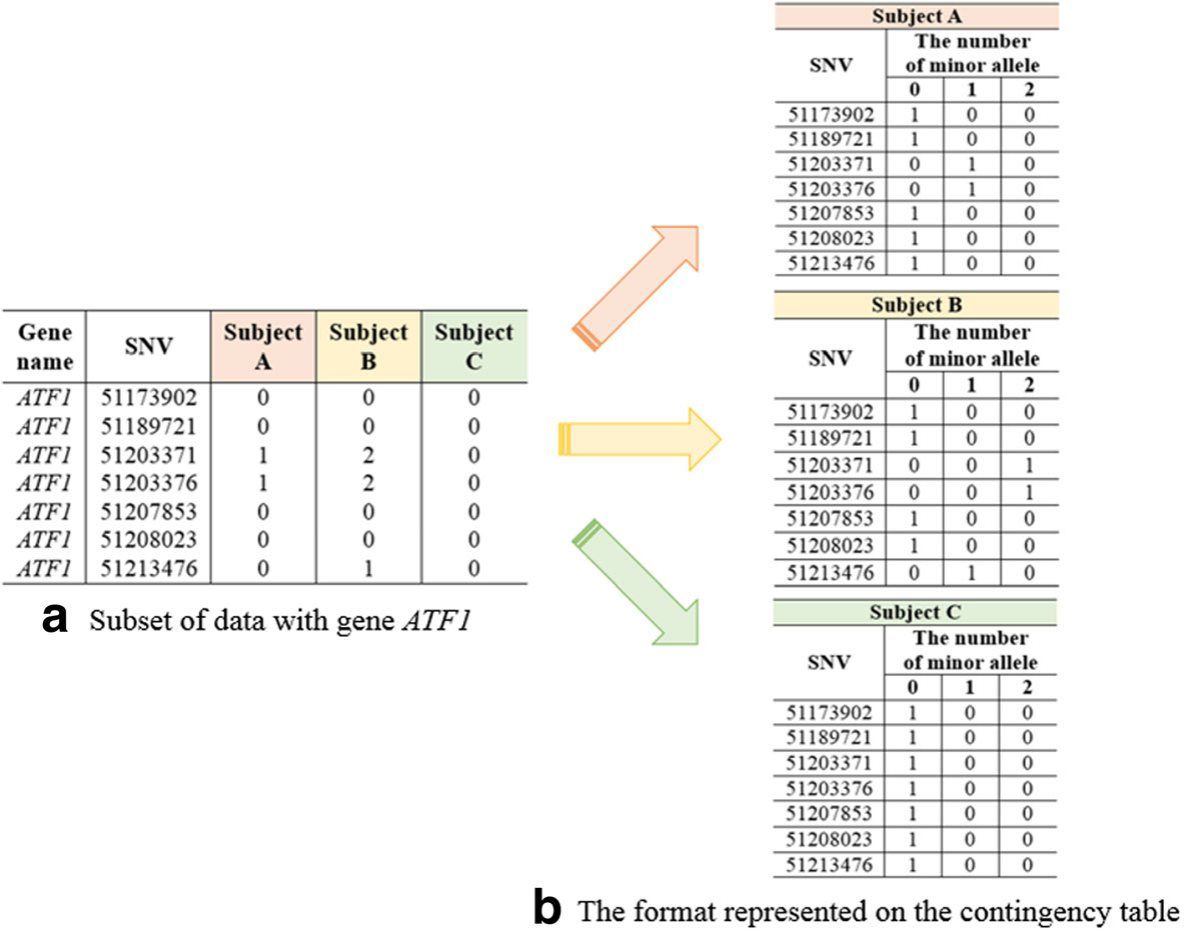
Transformation of a subset of data (**a**), into contingency tables (**b**), for three subjects, A, B, and C

For example, consider the gene *ATF1*, which contains seven SNVs in our IPMN dataset. Figure 1a shows the number of minor alleles for three subjects, A, B, and C. From these data, three 7 × 3 contingency tables could be constructed, as shown in Fig. 1b.

#### Generalized CMH statistic

Next, we recall the generalized CMH statistic to test the existence of partial association within the strata of the contingency table [10]. Here, we present it in a simpler form, specialized into types of contingency tables, as described in the previous subsection.

Let **n**_i_ = (*n*_*i*10_, *n*_*i*11_, ⋯, *n*_*it*2_)^′^ denote the (3*t*) × 1 vector of observed frequencies, let **n**_**ij.**_ = (*n*_*i*1._, *n*_*i*2._, ⋯, *n*_*it*._)^′^ denote the vector of the row marginal total number, and let **n**_**i.k**_ = (*n*_*i*. 0_, *n*_*i*. 1_, *n*_*i*. 2_)^′^ denote the vector of the column marginal totals, and let n_*i*. ._ denote the overall marginal total. Then, let *H*_0_ be the null hypothesis of no partial association, i.e., SNVs being independent of the number of minor alleles for a specific targeted gene. Note that all row marginal totals {**n**_**ij.**_} are 1, and n_*i*. ._ has the value *t* (i.e., the number of SNVs for each subject *i*). Hence, under *H*_0_, **n**_**i**_ (Table 2) follows the product’s multiple hypergeometric distribution, when the marginal totals are fixed, as in Fisher’s exact test.

$$ \mathbb{P}\left({\boldsymbol{n}}_i|{H}_0\right)=\frac{n_{i\bullet 0}!{n}_{i\cdotp 1}!{n}_{i\cdotp 2}!}{t!\prod \limits_{s=1}^t{n}_{is0}!{n}_{is1}!{n}_{is2}!}. $$

For the *i*th contingency table, define a t × 1 matrix ***P***_*i* ∗ ·_^′^ = (1, ⋯, 1)/t and a 3 × 1 matrix ***P***_*i* · ∗_^′^ = (*n*_*i* · 0_, *n*_*i* · 1_, *n*_*i* · 2_)/t. Denote ⊗ to be the Kronecker product defined for matrices, i.e., **A** ⊗ **B** = {*a*_*ij*_**B**} for **A** = {*a*_*ij*_}, and **B** of any dimension [13]. Then it is easy to check the following formulae for the mean and covariance of ***n***_*i*_**,** under *H*_0_:

$$ {\boldsymbol{m}}_i:= \mathbb{E}\left({\boldsymbol{n}}_i|{H}_0\right)=t\left[{\boldsymbol{P}}_{i\ast \cdotp}\otimes {\boldsymbol{P}}_{i\ast \cdotp}\right] $$

and

$$ \mathrm{Var}\left({\boldsymbol{n}}_i|{H}_0\right)=\frac{t^2}{t-1}\left[{\boldsymbol{D}}_{{\boldsymbol{P}}_{i\cdotp \ast }}-{\boldsymbol{P}}_{i\cdotp \ast }{\boldsymbol{P}}_{i\ast \cdotp}^{\prime}\right]\otimes \left[{\boldsymbol{D}}_{{\boldsymbol{P}}_{i\ast \cdotp }}-{\boldsymbol{P}}_{i\ast \cdotp }{\boldsymbol{P}}_{i\cdotp \ast}^{\prime}\right], $$

where for any vector ***v*** **=** (*v*_1_, ⋯, *v*_*k*_), ***D***_***v***_ denotes the diagonal matrix with *v*_*i*_ on its *i*th diagonal entry.

Since the degrees of freedom in our contingency table are 2(*t* − 1), we may eliminate the last column and row of each contingency table. For this purpose, let **A** = (**I**_t − 1_, **O**_t − 1_) ⊗ (**I**_2_, **O**_2_) be the matrix which eliminates the last row and column from each contingency table, where ***I***_*r*_ and ***O***_*r*_ denote the *r* × *r* identity matrix and the *r* × 1 matrix of 0’s, respectively. Let ***G***_*i*_ = *A*(***n***_*i*_ − ***m***_*i*_)). Denote ${\overset{\sim }{\boldsymbol{P}}}_{i\ast \cdotp }$ and ${\overset{\sim }{\boldsymbol{P}}}_{i\cdotp \ast }$ as the column vectors obtained by omitting the last entries of ***P***_*i* ∗ ·_**,**and ***P***_*i* · ∗_, respectively. Then, it is easy to verify that:

$$ \mathrm{Var}\left({G}_i|{H}_0\right)=\frac{t^2}{t-1}\left[{\boldsymbol{D}}_{{\boldsymbol{P}}_{i\cdotp \ast }}-{\overset{\sim }{\boldsymbol{P}}}_{i\cdotp \ast }{{\overset{\sim }{\boldsymbol{P}}}_{i\ast \cdotp}}^{\prime}\right]\otimes \left[{\boldsymbol{D}}_{{\boldsymbol{P}}_{i\ast \cdotp }}-{\boldsymbol{P}}_{i\ast \cdotp }{\boldsymbol{P}}_{i\cdotp \ast}^{\prime}\right]. $$

Now, the GCMH statistic for ***n***_*i*_

$$ \mathrm{GCMH}=\boldsymbol{G}{\left(\mathrm{Var}\left(\boldsymbol{G}|{H}_0\right)\right)}^{-1}{\boldsymbol{G}}^{\prime }, $$

where ***G*** =  ∑ ***G***_*i*_**.** It is well known that wth a large limit for *t*, the GCMH is asymptotically distributed as the chi-squared distribution, with degrees of freedom being 2(*t* − 1).

#### Exact association test (EXAT)

In this section, we propose a new statistic, which we call EXAT (*Exact Association Test*), to test the *difference* of partial association in two strata of contingency tables, corresponding to two groups, say, the case and control. The test statistic is simply the logarithmic ratio of the GCMH statistics computed for the two groups. Namely, denote the GCMH statistic of the case and control groups by CMH_case_,.and CMH_control_, respectively. Our proposed test statistic, T, is then defined by:

$$ \mathrm{T}=\log\ {\mathrm{CM}\mathrm{H}}_{\mathrm{case}}-\mathrm{logCM}{\mathrm{H}}_{\mathrm{control}}=\log \left(\frac{\mathrm{CM}{\mathrm{H}}_{\mathrm{case}}}{\mathrm{CM}{\mathrm{H}}_{\mathrm{control}}}\right). $$

Our motivation was as follows. In genetic association studies, we need to identify the genes associated with a certain phenotype of interest, such as disease status. Our assumption is that for the ‘causal’ genes, the case and control groups should show distinctive patterns of partial association. To measure this qualitative difference, we hypothesize that the intensity of partial association is proportional to the GCMH statistic. Hence, the more our test statistic T deviates from 0, the larger the partial associations between case and control groups.

This test statistic needs to be computed for each gene *X*. We then obtain *p*-values by a permutation procedure. Genes that have p-values smaller than the pre-specified significance level can be identified to associate with a disease status, e.g., in our current study, the progression of IPMN to pancreatic cancer.

#### Obtain *p*-values of EXAT using normal approximation

**Fig. 2:**
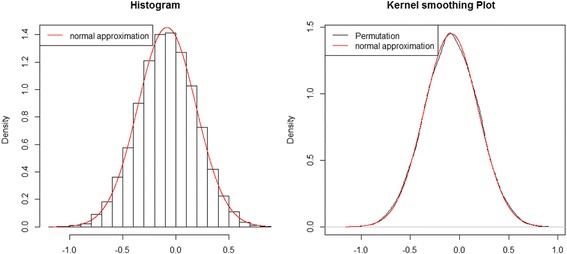
Histogram and kernel smoothed plot of test statistics

As the permutation test is computationally expensive, we considered an empirical but computationally efficient way to obtain p-values. We observed that our permuted test statistics were usually symmetric, with respect to 0, and followed a bell-shaped (i.e., normal) distribution. Moreover, it seemed that the distribution of T was closely approximated by a normal distribution, as determined by its first two moments. Figure 2 (left) shows a typical histogram of a randomly selected gene, generated by 10,000 permutations, having a normal distribution obtained by the first two moments in the histogram. Figure 2 (right) shows the kernel-smoothed plot of test statistics, which precisely agrees with a normal distribution. Based on this empirical evidence, we assert that T (the EXAT statistic) approximately follows a normal distribution.

**Fig. 3:**
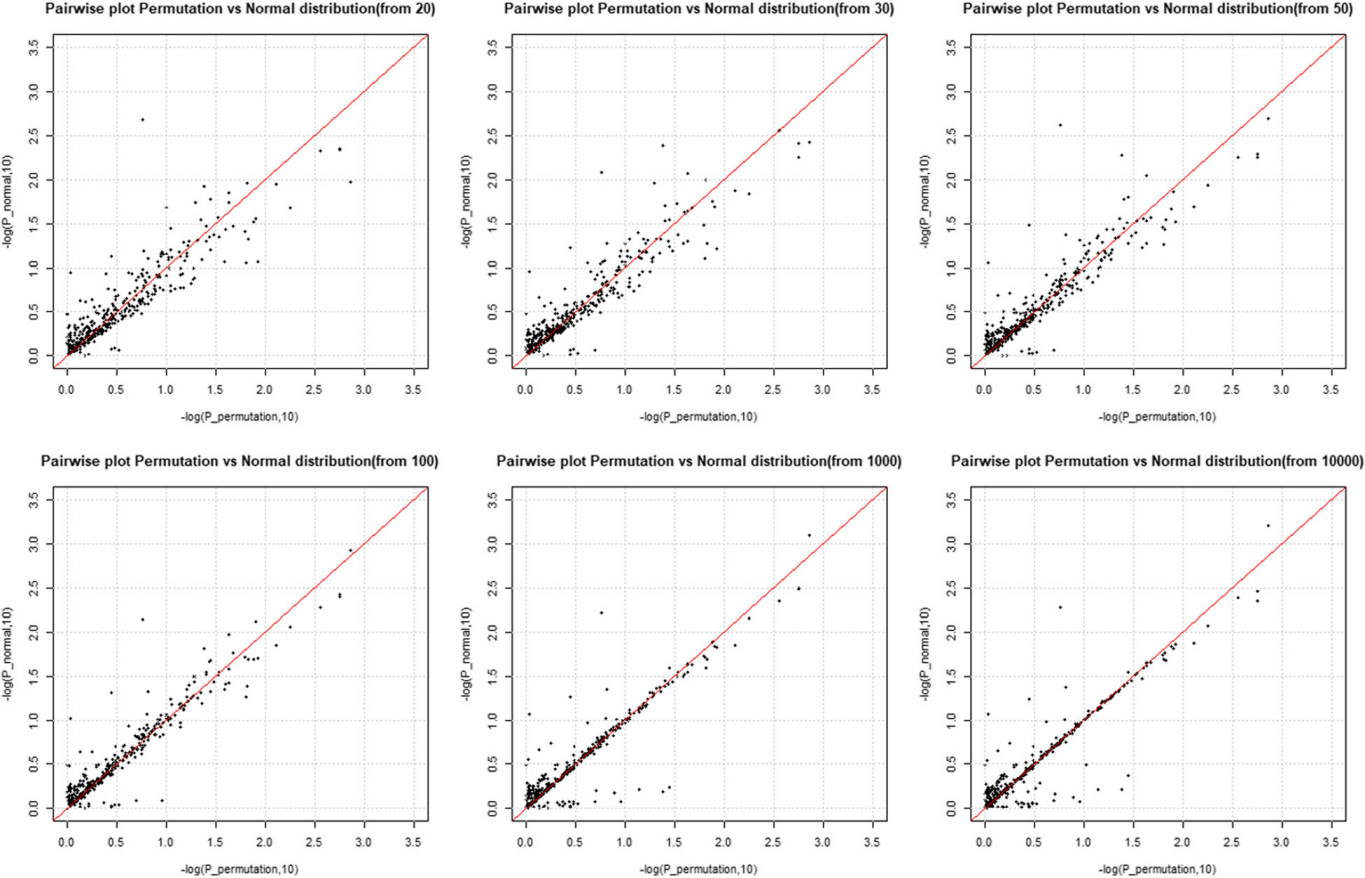
Pairwise *p*-value plots from permutations vs normal approximations, depending on the number of permutations

Based upon this observation, we decided to use the permutation method only to estimate the first two moments of T, and then use the resulting normal distribution, as an approximate distribution of T. Since we only need to estimate the first two moments of T for a normal approximation, we need many fewer permutations for normal approximation, but can also obtain similar results as the usual permutation method yields. Namely, as shown Fig. 3 and Table 3, we compared the *p*-values obtained from the distribution of T, as estimated by 10,000 permutations, with *p*-values obtained by normal approximation of the various number of permutations, ranging from 10 to 10,000. The resulting *p*-values of T, using the normal approximation, gave consistent results, with the usual permutation method. Furthermore, 20 permutations were sufficient enough (*R*^2^ > 0.8) to obtain similar *p*-values, from 10,000 permutations. Table 3 also shows the *p*-values from Kolmogorov-Smirnov test for comparing distributions, mean square errors, as well as two correlation coefficients [14]. All these results support the validity of normal approximation. Hence, we conclude that our hypothesized computational procedure of using a normal approximation not only gave consistent results, with the permutation test, but also was significantly reduced in computational burden.

**Table 3:** Measures from permutations, and normal approximation, depending on permutation times

Permutation times	20	30	50	100	1000	10,000
R^2^	82.0	85.4	85.1	89.0	85.2	84.9
Kolmogorov-Smirnov *p*-value	0.047	0.047	0.031	0.082	0.139	0.164
Mean square error	0.035	0.031	0.028	0.023	0.031	0.031
Pearson correlation	0.926	0.933	0.940	0.953	0.934	0.933
Spearman correlation	0.894	0.893	0.893	0.886	0.841	0.836

## Results

### Type I error simulations

We next performed a simulation study to validate our proposed method, EXAT. For this purpose, we generated simulated data, as in [7, 8], representing the sequence data of European population, from 4000 chromosomes, over 1 Mb regions, on the basis of a coalescent model that mimics the LD pattern local by using COSI [15]. We randomly selected 5-kb regions for testing for associations, under all simulation settings.

We generated datasets under the assumed null distribution, to evaluate the type I error control of EXAT. Dichotomous phenotypes, with 50% cases and 50% controls, were generated from a random sampling, under the null hypothesis.

We then applied EXAT to each randomly selected 5-kb regions. Then, we compared this result with the four of the most commonly used methods which are small-sample-adjusted SKAT (“SKAT”), small-sample-adjusted unified SKAT (“SKAT-O”), SKAT for the Combined Effect of Rare and Common Variants (“RC-SKAT”), and Burden test. We used the value of α =0.05, 0.01, and 0.001 under the five different total sample size settings (*n* = 50, 100, 200, and 500), with 4000 simulated datasets for each sample size. As shown in Table 4, EXAT had similar Type I error estimates regardless of sample size.

**Table 4:** Simulation studies of Type I Error estimates for the six different methods

	EXAT	SKAT	SKAT.O	RC-SKAT	Burden
*n* = 50					
α = 0.05	0.050	0.065	0.096	0.047	0.055
α = 0.01	0.011	0.018	0.020	0.008	0.005
α = 0.001	0.002	0.003	0.001	0.001	0.001
*n* = 100					
α = 0.05	0.044	0.062	0.073	0.050	0.055
α = 0.01	0.006	0.010	0.013	0.013	0.006
α = 0.001	0.000	0.001	0.003	0.001	0
*n* = 200					
α = 0.05	0.050	0.051	0.058	0.050	0.044
α = 0.01	0.011	0.010	0.011	0.012	0.009
α = 0.001	0.001	0.001	0.001	0.002	0.000
*n* = 500					
α = 0.05	0.045	0.049	0.047	0.040	0.046
α = 0.01	0.011	0.012	0.009	0.008	0.006
α = 0.001	0.002	0.001	0.000	0.001	0.001

### Real data application

We then applied the proposed EXAT to 395 cancer-associated genes. If any gene had only 1 SNV, we could not construct a contingency table for EXAT. In this case, we simply examined the significance of the association between disease status and the number of minor alleles, using Fisher’s exact test.

Through 10,000 permutations, our EXAT method identified 31 significant genes, at a significance level of 0.05 (Table 5), for four well-known oncogenes related to pancreatic cancer. Additionally, these four genes were each targeted at the beginning of the experiment. *P*-values from SKAT, SKAT-O, and RC-SKAT were obtained under adjustment for small samples. It is well known that mutations in *KRAS* are almost omnipresent in pancreatic cancer development and progression [16], and only our EXAT method could find *KRAS* as a significant gene.

**Table 5:** *P*-values from EXAT and competing methods, for the four targeted genes

Gene name	EXAT	SKAT	SKAT-O	RC-SKAT	Burden
KRAS	0.025	0.720	0.325	0.094	0.191
TP53	0.199	0.174	0.229	0.666	0.402
GNAS	0.597	0.426	0.597	0.405	0.988
CDH1	0.963	0.699	0.769	0.772	0.406

However, since the number of genes was large, compared to the small sample size, any significant gene was not detectable, through multiple comparison methods.

Table 6 shows 19 other genes known to be associated with pancreatic cancer [16–34]. For example, it has been reported that inhibition of PPP2R1A radiosensitizes pancreatic cancer via activation of CDC25C/CDK1, thus, PPP2R1A is a target gene for local therapy of pancreatic cancer [17]. The gene named *AURKB* is known to suppress proliferation of pancreatic cancer [18], and *KMT2D* is also known to be associated with pancreatic cancer [19, 20]. *MAPK1* is constitutively activated by frequent mutation and plays key roles in pancreatic carcinogenesis and progression [21]. Also, it has been reported that *FLT-1* is variably expressed in pancreatic cancer, and correlates significantly with disease stage [22]. It is also known that activation of the *PI3K* pathway mediates resistance to *MEK* inhibitors in *KRAS*-mutant cancers [23].

**Table 6:** *P*-values from EXAT, as compared to other methods, for identifying the significance of 19 pancreatic cancer-associated oncogenes

Gene name	EXAT	SKAT	SKAT-O	RC-SKAT	Burden
PPP2R1A	0.046	0.012	0.012	0.001	0.047
AURKB	0.002	0.059	0.001	0.019	0.029
CYP2C19	0.006	0.099	0.027	0.016	0.032
KMT2D	0.002	0.082	0.013	0.227	0.027
KRAS	0.025	0.720	0.325	0.094	0.191
PIK3C2B	0.029	0.377	0.025	0.452	0.036
CDH5	0.016	0.056	0.006	0.351	0.012
MAPK1	0.036	0.018	0.018	0.018	0.110
FLT1	0.001	0.148	0.120	0.105	0.108
PIK3CB	0.036	0.183	0.274	0.183	0.395
NBN	0.037	0.218	0.330	0.130	0.671
MSH6	0.015	0.161	0.214	0.119	0.872
LCK	0.042	0.250	0.082	0.250	0.099
ARID2	0.015	0.189	0.277	0.155	0.404
ADAMTS20	0.026	0.511	0.289	0.658	0.176
LPP	0.013	0.359	0.277	0.011	0.196
KDM6A	0.016	0.332	0.016	0.481	0.024
GUCY1A2	0.012	0.398	0.023	0.408	0.031
THBS1	0.049	0.108	0.165	0.077	0.377

**Fig. 4:**
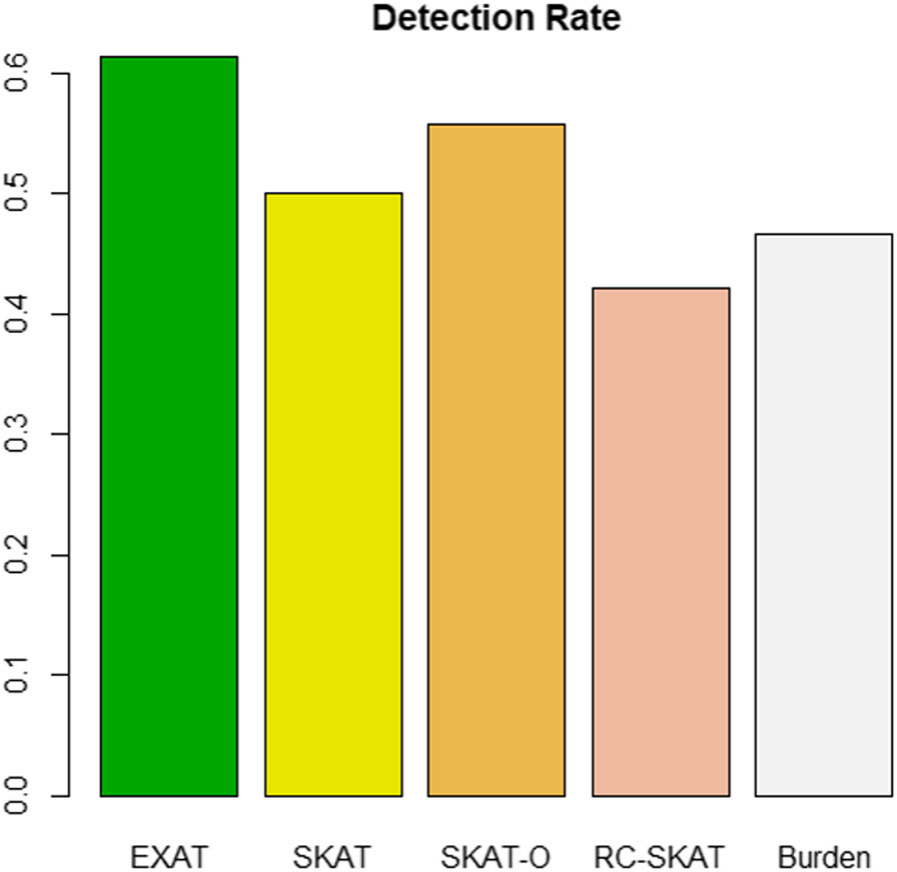
Detection Rate of EXAT and the competed methods

Figure 4 shows the detection rate for each method. Here, the detection rate is calculated as the ratio of the number of the genes reported to be associated with pancreatic cancer and the number of genes whose *p*-values are smaller than 0.05 [16–34]. EXAT has a better detection rate than other methods.

**Fig. 5:**
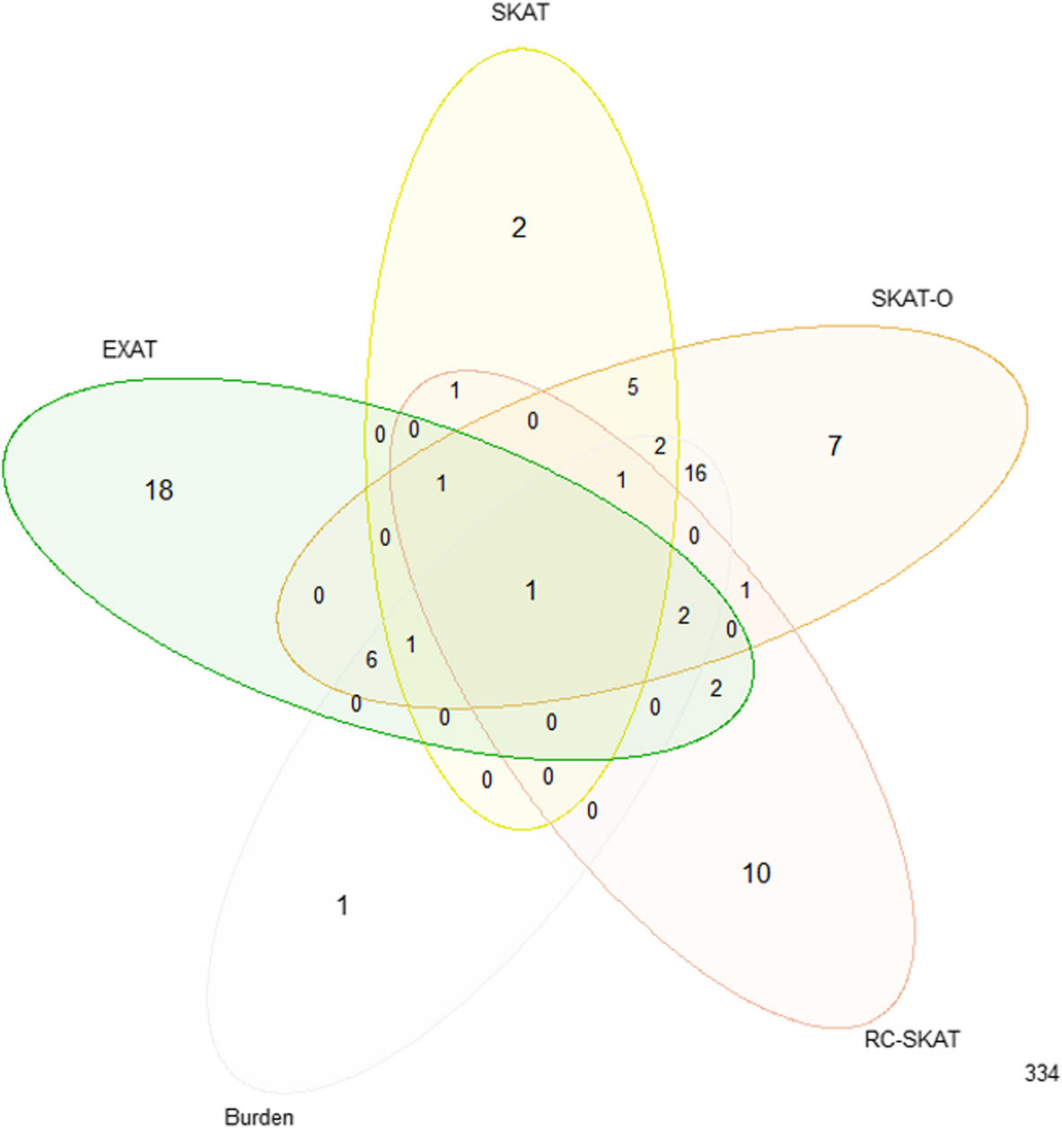
The Venn-diagram of the number of significant genes

Figure 5 shows a Venn diagram of the number of significant genes at a significance level of 0.05. Although all methods except RC-SKAT found *DDB2* as significant, the association of IPMN with these has not yet been experimentally verified.

**Fig. 6:**
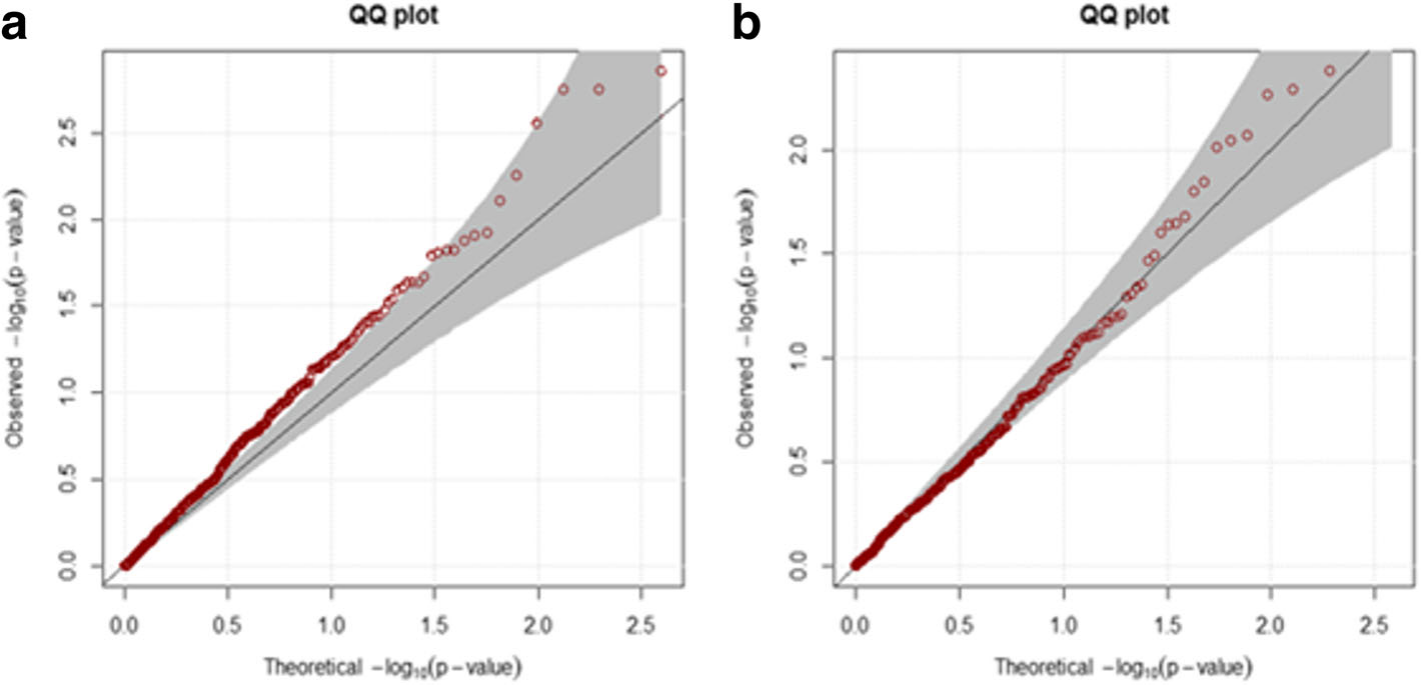
QQ plot of EXAT method. **a** A QQ plot from original data, and (**b**) A QQ plot from the data permuted disease status

A QQ plot of our EXAT method is shown in Fig. 6a, showing an inflation pattern. Since our NGS data was targeted, it contained many known or suspected oncogenes. In order to investigate whether the inflation was caused by association or false positives, we permuted the disease status (case and control) from our data, and then generated QQ plots. All QQ plots showed a similar pattern without any inflation. Figure 6b shows one representative QQ plot. Since there was no inflation after permutation, the inflation pattern in Fig. 6a was indeed due to genes causal to cancer.

**Fig. 7:**
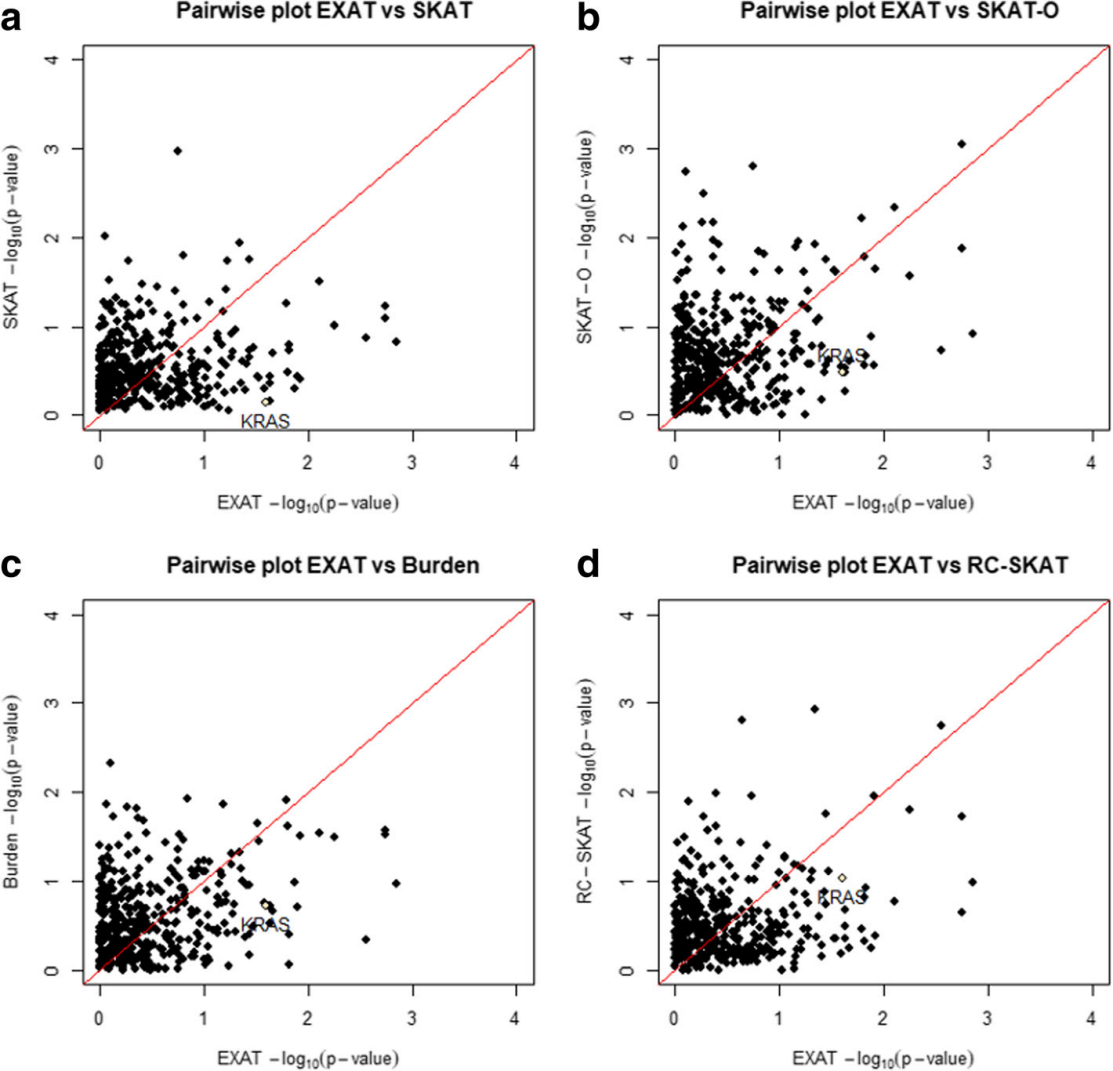
Comparison of pairwise scatter p-value plots of EXAT and other analysis methods. **a** EXAT vs SKAT, (**b**) EXAT vs SKAT-O, (**c**) EXAT vs Burden, and (**d**) EXAT vs RC-SKAT

Pairwise scatter plots of EXAT with SKAT, SKAT-O, Burden, and SKAT-RC, shown in Fig. 7, did not reveal any clear patterns.

## Discussion

As shown in Fig. 7, EXAT *p*-values differed from those of other methods, mainly because EXAT and other methods use different types of test statistics for detecting significant genes from NGS data. Our proposed EXAT uses the GCMH statistic for an array of contingency tables generated by the number of minor alleles and SNVs. Under the assumption of randomness within each group, EXAT is derived under a hypergeometric distributional assumption, conditioned by marginal totals. Thus, the ratio of two GCMHs, from case and control groups, is then used to compare the extent of partial association between case and control groups, and the p-values are obtained by permutation tests. On the other hand, burden tests aggregate information from all rare variants in a specific genomic region into a single summary variable, and obtain *p*-values through the chi-square distribution or Hoteling’s *t*-test. SKAT is based on a regression model, using a variance-component test to evaluate the significance of specific genes, using score test statistics, which follow the asymptotic chi-square distribution, under the null hypothesis.

In genetic association studies, individual covariate effects are often need to be adjusted for, although they are not of interest. Note that EXAT can handle any individual covariates of interest. Since EXAT was derived from the GCMH statistics from the subject specific contingency table given in Table 2, each contingency table is compared to its own hypergeometric means to obtain the GCMH statistics. As a result, each individual covariate effects are automatically adjusted for. When the interest lies in comparing a group effects such as gender, the stratified analysis can be applied.

Although EXAT uses a permutation procedure, it does not require a heavy computation time. In our IPMN data from SNUH consisting of 44 subjects with 8325 variants from 411 genes, it took 1.14 s to analyze the effect of single gene with 20 variants, which is the average gene size in IPMN data, using a standard desktop with a single processor Intel Core 2.5GHz CPU and 8GB RAM. For the entire analysis of total 411 genes, EXAT required 3 h for 1000 permutations. Alternatively, when we performed the EXAT test using normal approximation from 50 permutations, it took only 21 min, which demonstrates that the computational time could be substantially reduced when applying normal approximation.

Despite the superior performance of EXAT in distinguishing groups of different distributions, it does have the following limitations that warrant further improvement: (1) EXAT provides hypothesis testing results only; (2) EXAT may be insensitive when associations vary in direction (i.e., increase or decrease phenotypes) across all subjects within a group.

Lastly, in future studies, we will first compare the performance of EXAT with other existing tests for analyzing NGS data from small samples, using power simulation studies. Second, we can incorporate other types of GCMH statistics, such as mean score or correlation CMH, into our framework. The resulting test statistics may reflect further biological information, improving EXAT in terms of power. Lastly, we will also apply our method to the study of other NGS data in future research.

## Conclusions

In this study, we proposed an association test, *Exact Association Test* (EXAT), for identifying rare variants, and assessed its performance against other methods of analyzing small sample-size datasets associated with the intraductal papillary mucinous neoplasm (IPMN) subtype of pancreatic cancer. Thus, EXAT is an exact association test that does not require a large sample approximation. Our method is conceptually based upon Fisher’s exact test, and performs statistical analyses using the Generalized Cochran-Mantel-Haenszel (GCMH).

Since EXAT is valid for all sample sizes, it can be more accurate than SKAT in small sample studies, because SKAT relies on asymptotic tests, while EXAT does not. Indeed, as indicated in Fig. 7, among the five methods, only EXAT successfully identified *KRAS*, a well-known oncogene almost always mutated in pancreatic cancer [15]. This successful identification demonstrates that our newly proposed method can effectively identify cancer-susceptibility genes associated with the progression of IPMN to pancreatic cancer. We believe that our EXAT analyses will reveal rare but significant disease-associated oncogenes, and their constituent pathways, and thus increase our understanding of the etiology of cancer and other maladies.

## Abbreviations

CAST: Cohort allelic sum test
CMC: Combined multivariate and collapsing
CMH: Cochran-Mantel-Haenszel
EXAT: Exact Association Test
GCMH: Generalized Cochran-Mantel-Haenszel
GWAS: Genome-wide association studies
IPMN: Intraductal papillary mucinous neoplasm
MAF: Minor allele frequencies
NGS: Next generation sequencing
RC-SKAT: SKAT for the Combined Effect of Rare and Common Variants
SKAT: Sequence Kernel Association Test
SKAT-O: Unified SKAT
SNP: Single nucleotide polymorphism
SNV: Single nucleotide variant
VC: Variance-component
